# HIV Therapy: The Latest Developments in Antiviral Drugs—A Scoping Review

**DOI:** 10.3390/biomedicines13112629

**Published:** 2025-10-27

**Authors:** Francisco Fanjul, Meritxell Gavalda, Antoni Campins, Adria Ferré, Luisa Martín, María Peñaranda, Mari Ángeles Ribas, Elena Pastor-Ramon, Sophia Pinecki, Melchor Riera

**Affiliations:** 1 Infectious Diseases Unit, Hospital Universitario Son Espases, 07120 Palma de Mallorca, Spain; 2 Health Research Institute of the Balearic Islands (IdISBa), 07120 Palma de Mallorca, Spain; 3 Faculty of Medicine, University of the Balearic Islands, 07120 Palma de Mallorca, Spain; 4 Internal Medicine Department, Hospital de Inca, 07300 Inca, Spain; 5 Virtual Health Sciences Library of the Balearic Islands (Bibliosalut), 07007 Palma de Mallorca, Spain

**Keywords:** HIV, antiretroviral therapy, capsid inhibitor, long-acting therapy, maturation inhibitor, broadly neutralizing antibodies, prevention, implants, nanoparticles, scoping review

## Abstract

**Background:** Major advances in antiretroviral therapy (ART) have transformed HIV into a chronic condition, yet drug resistance, long-term toxicities, adherence challenges, and persistent viral reservoirs continue to drive innovation. **Objectives:** To map and synthesize recent developments in anti-HIV drugs and delivery platforms with a focus on (i) new molecules in clinical development and (ii) novel mechanisms of action, following a scoping review framework aligned with PRISMA-ScR. **Sources:** We interrogated PubMed, Embase.com, Web of Science, and Scopus (January 2020–September 2025) and screened abstracts from CROI, IAS/AIDS, IDWeek, and HIV Glasgow (2023–2025). **Content:** The evidence base underscores capsid inhibition (lenacapavir) for multidrug-resistant HIV and its expansion into prevention, long-acting intramuscular maintenance with cabotegravir/rilpivirine, maturation inhibitors (zabofiravir), and attachment inhibition with fostemsavir. Broadly neutralizing antibodies (bNAbs) can sustain ART-free suppression in selected individuals. Ultra-long-acting delivery systems are advancing toward translational evaluation. **Summary:** The pipeline is diversifying toward less frequent dosing, new targets, and combination strategies. Successful and ethical implementation will require resistance-informed selection, equitable access, and reimagined healthcare delivery models that accommodate long-acting technologies.

## 1. Introduction

The management of HIV has been revolutionized by antiretroviral therapy (ART), transforming a once-fatal infection into a manageable chronic condition. However, the paradigm of daily oral therapy, while highly effective, presents persistent challenges, including the cumulative burden of lifelong adherence, long-term toxicities, and the continuous threat of drug resistance [1]. This threat is driven by the intrinsic characteristics of HIV-1, including its high replication rate and the low fidelity of its reverse transcriptase enzyme, which introduces mutations with each replication cycle. The resulting viral quasispecies create a persistent reservoir of genetic diversity from which resistant variants can be selected under drug pressure, leading to virologic failure and limiting future therapeutic options. These challenges are not uniform and can be disproportionately burdensome for certain populations, including adolescents, individuals with comorbid mental health conditions, and those in resource-constrained settings where healthcare access is inconsistent. These limitations fuel the search for innovative therapeutic strategies.

In response, the field is undergoing a significant evolution. Two predominant themes are shaping the future of ART: the development of first-in-class molecules that engage novel viral targets, and the creation of novel drug delivery platforms designed to reduce dosing frequency [2]. Long-acting (LA) formulations, including injectable nanosuspensions and subcutaneous depots, aim to decouple therapeutic efficacy from the demands of daily adherence, a shift that could profoundly impact both treatment and prevention [3].

This scoping review provides a comprehensive map of these recent developments. We focus on compounds that have reached clinical phases, synthesizing pivotal trial outcomes and mechanistic innovations. By examining the emerging landscape of capsid inhibitors, maturation inhibitors, broadly neutralizing antibodies, and ultra-long-acting delivery systems, this review aims to contextualize the next generation of ART and anticipate its clinical and public health implications.

## 2. Methods

We conducted a scoping review, following the methodological principles outlined by Arksey and O’Malley [4] and aligning our reporting with the PRISMA Extension for Scoping Reviews (PRISMA-ScR) [5]. As a scoping review, the aim is to map the breadth of emerging evidence rather than to conduct a formal quality appraisal or quantitative synthesis of trial results. Our selection criteria prioritized agents that have entered clinical development (Phase 1 or later) or preclinical platforms with novel mechanisms and significant proof-of-concept, ensuring a focus on innovations with near-to-mid-term clinical relevance.

### Search Strategy and Selection

**Figure 1:**
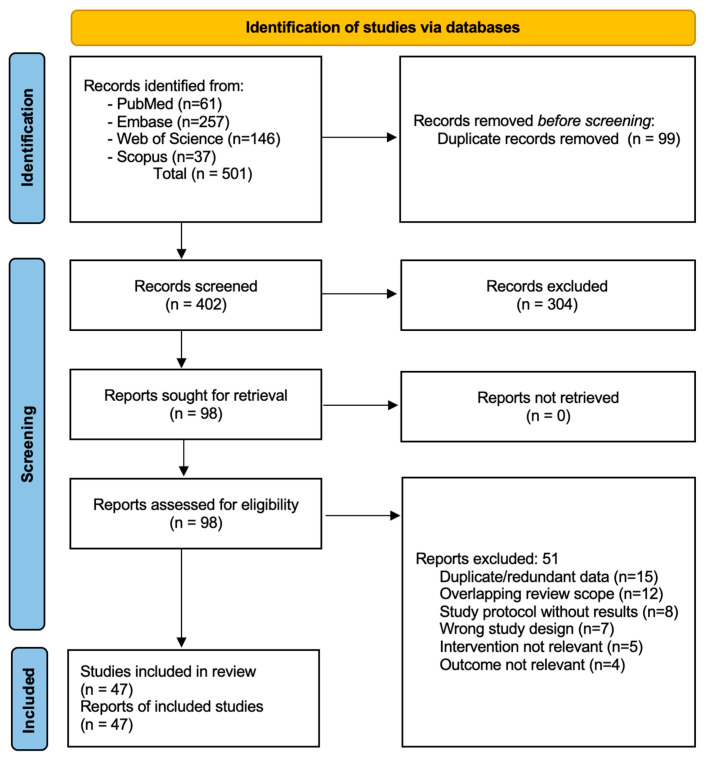
PRISMA-ScR Flow Diagram.

A systematic search was designed and executed by a health sciences librarian (E.P.-R.). We searched PubMed, Embase.com, Web of Science, and Scopus for records published between January 2020 and September 2025. The detailed search strategies are provided in the Supplementary Material (Table S1). Supplementary Table S1 details the specific search terms, Boolean operators, and filters used for each of the four databases. The initial search yielded 501 records. After removing 99 duplicates, 402 unique articles were screened based on their title and abstract, which resulted in the exclusion of 304 records. The full texts of the remaining 98 articles were retrieved and assessed for eligibility. Of these, 51 were excluded. A final selection of 47 studies meeting the inclusion criteria was identified through the systematic search. The characteristics of these 47 included studies are summarized in the Supplementary Material (Table S2). Supplementary Table S2 provides a structured summary of each included study, detailing the author, study design, intervention, population, and key outcomes. The study selection process is detailed in the PRISMA flow diagram (Figure 1). Additional key sources, such as clinical guidelines, were included in the final reference list based on expert review.

## 3. Results

The results of our review highlight a rapidly evolving pipeline of HIV therapeutics. For clarity, we have categorized the findings by mechanism of action and summarized key agents in Table 1.

### 3.1. Capsid Inhibition: Lenacapavir

Lenacapavir (LEN) represents the first-in-class HIV-1 capsid inhibitor, a novel mechanism that targets multiple stages of the viral lifecycle. By binding to a conserved pocket at the interface of capsid protein (p24) monomers, LEN allosterically disrupts the delicate processes of viral core assembly (a late-stage event) and disassembly (an early-stage event). This dual mechanism confers picomolar potency and activity against all major HIV-1 subtypes, with no cross-resistance to existing antiretroviral classes [6].

The pivotal evidence for LEN in treatment comes from the CAPELLA trial, a Phase 2/3 study in heavily treatment-experienced (HTE) individuals with multidrug-resistant virus. In the randomized cohort, adding oral LEN to a failing regimen for 14 days resulted in a ≥ 0.5 log10 copies/mL viral load reduction in 88% of participants, compared to only 17% in the placebo group [7]. After this functional monotherapy period, participants switched to an optimized background regimen (OBR) plus subcutaneous LEN every 26 weeks. At week 52, 83% of participants achieved virologic suppression (HIV-1 RNA <50 copies/mL) [7]. These high rates of suppression were durable, with 82% of those remaining on study maintaining suppression at week 104, accompanied by a robust mean increase in CD4 count of 122 cells/µL [8].

The unique pharmacokinetic profile of LEN, characterized by a very long half-life, enables its semi-annual subcutaneous dosing. Studies have also confirmed that an oral dose of 300 mg once weekly can be used effectively as a bridging therapy if a subcutaneous injection is missed [9]. Furthermore, a new intramuscular formulation has shown potential for once-yearly administration [10].

Resistance to LEN is primarily associated with mutations in the Gag protein at or near the capsid binding site, such as M66I [7,11]. While highly effective, the relatively low genetic barrier to resistance for lenacapavir is a key clinical consideration. A low genetic barrier implies that a small number of viral mutations, sometimes just a single nucleotide change, can confer a significant reduction in susceptibility to the drug. For lenacapavir, several key mutations (e.g., M66I, Q67H, K70N) have been identified that can reduce its activity [11]. The emergence of resistance during functional monotherapy in trials underscores the critical need for it to be paired with other active agents, especially given its long pharmacokinetic tail, which can create extended periods of low-level drug exposure if adherence is suboptimal or if a follow-up dose is missed. This long tail is a double-edged sword: while it provides forgiveness for slightly delayed doses, a significantly late or missed dose can result in a prolonged period of sub-therapeutic drug concentrations, an ideal scenario for the selection and amplification of pre-existing resistant variants. Recent analyses from clinical trials have further characterized these resistance pathways, emphasizing that the risk of resistance emergence is highest when lenacapavir is not combined with at least one other fully active agent in the regimen [12]. In vitro studies have shown no cross-resistance between LEN and other antiretroviral classes, including entry inhibitors, underscoring its value in salvage therapy [13].

Beyond treatment, LEN has demonstrated transformative potential in HIV prevention (PrEP). The PURPOSE 1 and 2 trials showed superior efficacy of semi-annual subcutaneous LEN over daily oral emtricitabine/tenofovir alafenamide (F/TAF) [14,15]. These landmark results led the FDA to grant LEN a Breakthrough Therapy designation for PrEP.

### 3.2. Long-Acting Intramuscular Maintenance: Cabotegravir/Rilpivirine

The combination of cabotegravir (CAB), an integrase inhibitor, and rilpivirine (RPV), a non-nucleoside reverse transcriptase inhibitor, was the first complete long-acting injectable regimen approved. The non-inferiority of monthly injections versus daily oral therapy was established in the pivotal Phase 3 ATLAS and FLAIR trials [16,17]. The ATLAS-2M trial subsequently demonstrated that dosing every two months (Q2M) was also non-inferior to monthly (Q1M) dosing through 152 weeks [18,19]. Patient-reported outcomes from ATLAS-2M showed high treatment satisfaction and a strong preference for the Q8W dosing schedule [20].

The primary clinical challenge is managing virologic failure, with predictors including baseline RPV resistance (particularly mutations like K101E or E138K), HIV-1 subtype A6/A1, and high BMI [21,22]. Emerging real-world data also highlight the risk of developing cross-class resistance, including to integrase inhibitors (e.g., Q148R or N155H), in the small number of individuals who experience virologic failure, making subsequent treatment options more complex [22]. The development of resistance to both components of the injectable regimen is a major concern, as it can compromise the efficacy of entire drug classes. For instance, the emergence of INSTI resistance could preclude the use of highly effective future regimens based on second-generation integrase inhibitors like dolutegravir or bictegravir. This underscores the importance of careful patient selection, including baseline resistance testing as recommended by treatment guidelines. Furthermore, final data from large-scale prevention trials using long-acting cabotegravir have shown that while highly effective, rare breakthrough HIV infections can occur, sometimes leading to the development of integrase inhibitor resistance, which reinforces the critical need for frequent HIV screening during PrEP roll-out [23]. A major advancement in implementation has been the CARES trial, which demonstrated non-inferiority and safety of Q8W CAB-RPV LA in several African countries [24]. This trial was critical in providing evidence for the feasibility and acceptability of this regimen in resource-limited settings, a key step toward global access. An ultra-long-acting formulation (CAB-ULA) is also in development, with Phase 1 data suggesting potential for dosing every four months or longer [25].

### 3.3. Attachment Inhibition: Fostemsavir (Ftr)

Fostemsavir (FTR) is a first-in-class attachment inhibitor and a prodrug of temsavir, which binds directly to the HIV-1 gp120 surface protein, locking it in a closed conformation and preventing viral attachment to the host cell’s CD4 receptor [26]. Its efficacy in HTE patients was demonstrated in the BRIGHTE study, where 60% of participants achieved virologic suppression at week 96, with durable immunologic recovery sustained through 240 weeks [27,28]. Participants also reported significant improvements in quality of life [29], and sub-studies showed reductions in biomarkers of inflammation and coagulopathy [30].

### 3.4. Maturation Inhibitors: Zabofiravir (Gsk3640254)

Maturation inhibitors target the final step of Gag polyprotein cleavage. Zabofiravir (GSK3640254) is a second-generation agent with potent activity across diverse HIV-1 subtypes and baseline Gag polymorphisms [31]. A Phase IIa monotherapy study confirmed its dose-dependent antiviral activity [32], and it has shown no clinically significant pharmacokinetic interaction when co-administered with dolutegravir [33]. Resistance to this class typically involves mutations in Gag at the cleavage site between capsid (p24) and spacer peptide 1 (SP1). A key advantage of this mechanism is its novel resistance profile, which is not expected to show cross-resistance with existing antiretroviral classes, making it a potentially important component of future combination regimens for both treatment-naïve and experienced individuals. For HTE patients who have accumulated extensive resistance mutations to protease inhibitors, NRTIs, NNRTIs, and even integrase inhibitors, the availability of a new class with a completely distinct resistance pathway is of paramount clinical value. It offers a new tool to construct a viable, suppressive regimen where options were previously exhausted.

### 3.5. Broadly Neutralizing Antibodies (Bnabs)

Broadly neutralizing antibodies (bNAbs) are a form of immunotherapy for HIV [34]. They are selected for clinical development based on their potency and breadth, with combinations chosen to target distinct, non-overlapping epitopes on the viral envelope protein. This multi-target strategy creates a higher barrier to resistance, forcing the virus to mutate at several sites to escape neutralization. The HIV envelope is highly variable, and the virus can rapidly mutate to escape the pressure of a single antibody. However, by combining two or three bNAbs that target conserved regions—for example, the CD4 binding site, the V3 loop, and the MPER region—the virus would need to accumulate multiple simultaneous mutations to evade neutralization, a far less probable event. This strategy mimics the principles of combination ART. Combinations of two or three bNAbs have demonstrated the ability to maintain virologic suppression in individuals who pause ART [35,36,37], though success is highly dependent on baseline viral sensitivity [38]. Indeed, overcoming the challenges of pre-existing viral resistance and subsequent viral escape remains a primary hurdle for bNAb therapy, requiring the development of more potent and broader antibody combinations or engineered antibodies to increase the barrier to resistance [39]. Their role in cure strategies when combined with latency-reversing agents (LRAs) has shown modest results in trials like TITAN (LRA lefitolimod) and ROADMAP (LRA romidepsin), where no significant additional benefit was observed [40,41]. For prevention, the AMP trials provided proof-of-concept that bNAbs can prevent acquisition of sensitive HIV strains [42]. The future lies in improved combinations, including long-acting drugs like lenacapavir [43].

### 3.6. Weekly Oral Regimens: Islatravir

Islatravir (ISL) is a potent nucleoside reverse transcriptase translocation inhibitor (NRTTI) with a long intracellular half-life [44]. Early-phase studies showed promising efficacy [45]. However, development of the 0.75 mg daily dose was halted due to observations of dose-dependent decreases in total lymphocyte and CD4 T-cell counts [46,47]. The program is now moving forward with a lower dose.

### 3.7. Other Dosing Strategies

The ANRS 170 QUATUOR trial successfully demonstrated that a maintenance strategy of taking oral ART for four consecutive days followed by three days off was non-inferior to continuous daily therapy over 48 weeks [48].

### 3.8. Ultra-Long-Acting Delivery Systems and Cure-Adjacent or Preclinical Cure-Adjacent Strategies

Beyond injectables, research into novel delivery platforms is advancing. In preclinical stages, in situ forming implants (ISFIs) that provide months-long release of cabotegravir have protected macaques from SHIV infection [49]. Receptor-targeted nanoparticles are also being explored in laboratory models to deliver drugs to sanctuary sites.

In cure research, latency-reversing agents like the TLR-7 agonist vesatolimod have shown modest delays in viral rebound in clinical trials [50], while “block-and-lock” strategies using agents like didehydro-cortistatin A suppress rebound in preclinical models [51].

## 4. Discussion

This scoping review charts a clear and exciting trajectory for the future of HIV therapy. The pipeline is robust, characterized by a dual focus on diversifying mechanisms of action and dramatically reducing dosing frequency. The approval and successful implementation of CAB-RPV LA have provided the real-world proof-of-concept for long-acting injectable therapy. The development of agents like lenacapavir represents a monumental leap forward and a significant advance, bridging the gap between treatment and prevention. The emergence of novel mechanisms is fundamentally changing the approach to multidrug-resistant HIV.

However, these innovations are accompanied by significant implementation challenges. Long-acting therapies require a paradigm shift in healthcare delivery, moving from a patient-managed, pharmacy-centric model to a clinician-administered, systems-dependent one. This necessitates innovations in supply chain management, patient scheduling and tracking through electronic health records, and developing robust re-engagement strategies for individuals who miss appointments. Resistance management becomes more complex due to the long pharmacokinetic tail of these drugs. A prolonged, low-level drug concentration following a missed dose can facilitate the selection of resistant mutations, making adherence to the injection schedule even more critical than with daily oral therapy. Standardized protocols for resistance testing, oral bridging, and patient re-engagement are critical to steward these new technologies effectively, as reflected in current treatment guidelines [52,53,54,55]. Furthermore, staying updated with the latest consensus on resistance mutations is essential for clinical practice [11].

A deeper dive into resistance management reveals several layers of complexity. The first is the aforementioned issue of the long pharmacokinetic tail. For drugs like injectable cabotegravir, rilpivirine, and lenacapavir, plasma concentrations can persist for months to over a year after the last dose. During this extended period, drug levels will inevitably fall below the inhibitory concentration required for full viral suppression. If a patient disengages from care, this creates a prolonged window of functional monotherapy or dual therapy at suboptimal doses, which is a potent driver for the selection of resistance. This has significant implications for clinical practice, requiring robust strategies for oral lead-in or oral bridging therapies to cover planned (or unplanned) interruptions in injections.

Secondly, the challenge of resistance testing itself, particularly in LMICs, cannot be overstated, a point raised by our reviewers. Standard genotypic resistance testing requires sophisticated laboratory infrastructure, trained personnel, and significant financial investment, all of which are scarce in many high-burden settings. Furthermore, interpreting the results requires expertise. While simplified and lower-cost assays are in development, their widespread availability remains a future goal. Without access to baseline resistance testing, clinicians are unable to identify individuals with pre-existing resistance (e.g., to NNRTIs like rilpivirine) who are at high risk of failure on long-acting injectable regimens. This can lead to the unwitting prescription of ineffective therapy and the further amplification and transmission of drug-resistant HIV, undermining both individual and public health.

A third, more insidious challenge is that of archived resistance. HIV integrates its genetic material into the host cell’s genome, creating a long-lived latent reservoir of proviral DNA. Resistance mutations acquired years or even decades earlier can persist silently within this reservoir. Standard clinical resistance tests only analyze the viral strains actively circulating in the blood plasma. Therefore, a patient may appear to have a fully susceptible virus, but archived mutations can re-emerge upon initiation of a new therapy to which they confer resistance. This is particularly relevant for HTE patients, whose viral reservoirs may harbor a complex history of resistance mutations. New agents must not only be active against the circulating virus but must also ideally have a high barrier to resistance to prevent the rapid selection of these archived variants.

Most importantly, ensuring equitable access is paramount. There is a significant risk that these advanced, more expensive therapies will exacerbate existing health disparities, creating a two-tiered system of care. The populations who stand to benefit most—including marginalized groups with adherence challenges and those in resource-limited settings—are often the last to access biomedical innovations. The successful rollout demonstrated in the CARES trial in Africa is a promising step, but it must be scaled up. Operationalizing access in LMICs presents formidable hurdles, including the high cost of the drugs and the lack of infrastructure for resistance testing, which is often recommended before initiating long-acting therapies. Actionable strategies are needed, such as tiered pricing, technology transfers, task-shifting injection administration to nurses or community health workers, and developing low-cost, point-of-care diagnostics to guide patient selection. A concerted global effort will be required to ensure that these transformative therapies reach those who need them most. Finally, as the field moves toward immunotherapies and potentially gene-based strategies, new ethical and regulatory challenges will emerge, including the complexity of informed consent for novel interventions and the need for long-term safety and efficacy monitoring.

## 5. Limitations

As a scoping (rather than systematic) review, this work emphasizes breadth over exhaustive critical appraisal; meta-analysis was not attempted. Our methodology was designed to map the field, not to provide definitive clinical recommendations based on a quantitative synthesis of evidence. Several promising findings derive from conference abstracts or preclinical studies; conclusions may evolve with peer review and phase 3 results.

## 6. Conclusions

Next-generation HIV therapy is converging on less frequent dosing and novel mechanisms that expand options across treatment and prevention. Translational success will hinge on resistance-informed patient selection, operational readiness for long-acting services, and an unwavering commitment to inclusive access.

## Figures and Tables

**Table 1:** Summary of Novel HIV Therapeutic Agents in Development.

Mechanism of Action	Agent(s)	Phase of Development	Dosing Frequency
Capsid Inhibition	Lenacapavir (LEN)	Approved/Phase 3 (PrEP)	Subcutaneous every 6 months
Integrase + NNRTI	Cabotegravir/Rilpivirine (CAB/RPV LA)	Approved	Intramuscular every 1–2 months
Attachment Inhibition	Fostemsavir (FTR)	Approved	Oral, twice daily
Maturation Inhibition	Zabofiravir (GSK3640254)	Phase 2b	Oral, daily
Immunotherapy	Broadly Neutralizing Antibodies (bNAbs)	Phase 2/3	Intravenous/Subcutaneous (variable)
NRTTI	Islatravir (ISL)	Phase 3 (lower dose)	Oral, weekly (investigational)

PrEP: pre-exposure prophylaxis, NNRTIs: Non-nucleoside reverse transcriptase inhibitors.

## Data Availability

Not applicable.

## Supplementary Materials

The following supporting information can be downloaded at: https://www.mdpi.com/article/10.3390/biomedicines13112629/s1, Table S1: Search strategy; Table S2: Characteristics of the included studies.
