# Supplementary material for: HIV Therapy: The Latest Developments in Antiviral Drugs—A Scoping Review
Source: Biomedicines. 2025 Oct 27;13(11):2629. doi: 10.3390/biomedicines13112629 (PMC12650496; doi:10.3390/biomedicines13112629)
Supplement: Supplementary file 1 [file biomedicines-13-02629-s001.zip › biomedicines-3906926-supplementary.pdf]

Table S1. Detailed Search Strategies

Database	Strategy	Results
PubMed	((""Antiretroviral Therapy""[Title/Abstract] AND (""HIV Integrase Inhibitors""[Title/Abstract] OR ""Integrase Inhibitors""[Title/Abstract]) AND (""lenacapavir""[Title/Abstract] OR ""cabotegravir""[Title/Abstract] OR ""cabotegravir, rilpivirine drug combination""[Title/Abstract] OR ""Rilpivirine""[Title/Abstract] OR ""fostemsavir""[Title/Abstract] OR ""GSK3640254""[Title/Abstract] OR ""islatravir""[Title/Abstract])) OR (""maturation inhibitors""[Title/Abstract] OR ""capsid inhibitor""[Title/Abstract] OR ""MK-8527""[Title/Abstract] OR ""Broadly Neutralizing Antibodies""[Title/Abstract]) AND (""HIV""[Title/Abstract])) OR ((((""Antiretroviral Therapy, Highly Active""[Mesh]) AND (""HIV Integrase Inhibitors""[Mesh] OR ""Integrase Inhibitors""[Mesh]) AND (""lenacapavir""[Supplementary Concept] OR ""cabotegravir""[Supplementary Concept] OR ""cabotegravir, rilpivirine drug combination""[Supplementary Concept] OR ""Rilpivirine""[Mesh] OR ""fostemsavir""[Supplementary Concept] OR ""GSK3640254""[Supplementary Concept] OR ""islatravir""[Supplementary Concept])) OR (""maturation inhibitors""[Title/Abstract] OR ""capsid inhibitor""[Title/Abstract] OR ""MK-8527""[Title/Abstract] OR ""Broadly Neutralizing Antibodies""[Mesh])) AND (""HIV""[Mesh])) AND ((clinicaltrial[Filter] OR clinicaltrialphasei[Filter] OR clinicaltrialphaseii[Filter] OR clinicaltrialphaseiii[Filter] OR clinicaltrialphaseiv[Filter] OR clinicaltrialprotocol[Filter] OR controlledclinicaltrial[Filter] OR systematicreview[Filter]) AND (2020:2025[pdat]))	61
Embase	('human immunodeficiency virus'/exp AND 'antiretroviral therapy'/exp AND ('long acting drug'/exp OR 'integrase inhibitor'/exp OR 'lenacapavir'/exp OR 'cabotegravir'/exp OR 'rilpivirine'/exp OR 'fostemsavir'/exp OR 'fipravirimat'/exp OR 'broadly neutralizing antibody'/exp OR 'implant'/exp OR 'nanoparticle'/exp OR 'islatravir'/exp)) OR (('hiv':ab,ti OR 'human immunodeficiency virus':ab,ti) AND 'antiretroviral therapy':ab,ti AND ('long acting drug':ab,ti OR 'integrase inhibitor':ab,ti OR 'lenacapavir':ab,ti OR 'cabotegravir':ab,ti OR 'rilpivirine':ab,ti OR 'fostemsavir':ab,ti OR 'fipravirimat':ab,ti OR 'broadly neutralizing antibody':ab,ti OR 'implant':ab,ti OR 'nanoparticle':ab,ti OR 'islatravir':ab,ti OR 'mk-8527':ab,ti OR 'capsid inhibitor':ab,ti OR 'maturation inhibitors':ab,ti OR 'zabofiravir':ab,ti)) AND [2020-2025]/py AND ('clinical trial'/de OR 'phase 3 clinical trial'/de OR 'randomized controlled trial topic'/de OR 'systematic review'/de)	257
Web of Science	((TS=((""HIV"" OR ""human immunodeficiency virus"") AND ""antiretroviral therapy"" AND (""long-acting"" OR ""integrase inhibitors"" OR ""lenacapavir"" OR ""cabotegravir"" OR ""rilpivirine"" OR ""fostemsavir"" OR ""maturation inhibitors"" OR ""zabofiravir"" OR ""GSK3640254"" OR ""broadly neutralizing antibodies"" OR ""implants"" OR ""nanoparticles"" OR ""capsid inhibitor"" OR ""MK-8527"" OR ""islatravir""))) OR AB=((""HIV"" OR ""human immunodeficiency virus"") AND ""antiretroviral therapy"" AND (""long-acting"" OR ""integrase inhibitors"" OR ""lenacapavir"" OR ""cabotegravir"" OR ""rilpivirine"" OR ""fostemsavir"" OR ""maturation inhibitors"" OR ""zabofiravir"" OR ""GSK3640254"" OR ""broadly neutralizing antibodies"" OR ""implants"" OR ""nanoparticles"" OR ""capsid inhibitor"" OR ""MK-8527"" OR ""islatravir""))) OR TI=((""HIV"" OR ""human immunodeficiency virus"") AND ""antiretroviral therapy"" AND (""long-acting"" OR ""integrase inhibitors"" OR ""lenacapavir"" OR ""cabotegravir"" OR ""rilpivirine"" OR ""fostemsavir"" OR ""maturation inhibitors"" OR ""zabofiravir"" OR ""GSK3640254"" OR ""broadly neutralizing antibodies"" OR ""implants"" OR ""nanoparticles"" OR ""capsid inhibitor"" OR ""MK-8527"" OR ""islatravir""))	146
Scopus	TITLE-ABS-KEY (('HIV' OR 'human immunodeficiency virus') AND 'antiretroviral therapy' AND ('long-acting' OR 'integrase inhibitors' OR 'lenacapavir' OR 'cabotegravir' OR 'rilpivirine' OR 'fostemsavir' OR 'maturation inhibitors' OR 'zabofiravir' OR	37

	'GSK3640254' OR 'broadly neutralizing antibodies' OR 'implants' OR 'nanoparticles' OR 'capsid inhibitor' OR 'MK-8527' OR 'islatravir')) AND PUBYEAR > 2019 AND PUBYEAR < 2026	

Table S2. Characteristics of Included Studies

Author (Year)	Study Design	Intervention	Population	Key Outcome
CAPSID INHIBITORS				
Segal-Maurer (2022) [7]	Phase 2/3, RCT	Lenacapavir + OBR	HTE with MDR-HIV	High viral suppression rate (83% <50 copies) at week 52.
Ogbuagu (2025) [8]	Phase 2/3, follow-up	Lenacapavir + OBR	HTE with MDR-HIV	Durable viral suppression (82% <50 copies) at week 104.
Ogbuagu (2025) [9]	Post-hoc analysis	Oral lenacapavir (bridging)	PWH on SC LEN	Oral bridging maintained viral suppression and was well-tolerated.
Jogiraju (2025) [10]	Phase 1, open-label	IM lenacapavir (annual)	Healthy volunteers	PK profile supports a once-yearly dosing schedule.
Margot (2023) [12]	Resistance analysis	Lenacapavir	CAPELLA participants	No cross-resistance observed with entry inhibitors.
Bekker (2024) [13]	Phase 3, RCT (PURPOSE 1)	SC lenacapavir (PrEP)	Cisgender women	100% efficacy in preventing HIV acquisition.
Mayer (2025) [14]	Phase 3, RCT (PURPOSE 2)	SC lenacapavir (PrEP)	MSM and trans women	Superior efficacy compared to daily oral PrEP.
INTEGRASE + NNRTI (LONG-ACTING)				
Swindells (2020) [15]	Phase 3, RCT (ATLAS)	Monthly CAB+RPV LA	Virologically suppressed adults	Non-inferior to daily oral ART for maintaining suppression.
Orkin (2020) [16]	Phase 3, RCT (FLAIR)	Monthly CAB+RPV LA	Virologically suppressed adults	Non-inferior to daily oral ART after DTG-based induction.
Overton (2020) [17]	Phase 3b, RCT (ATLAS-2M)	CAB+RPV LA Q8W vs Q4W	Virologically suppressed adults	Q8W dosing was non-inferior to Q4W at week 48.

Overton (2023) [18]	Phase 3b, follow-up (ATLAS-2M)	CAB+RPV LA Q8W vs Q4W	Virologically suppressed adults	Non-inferiority was maintained through week 152.
Chounta (2021) [19]	PRO analysis (ATLAS-2M)	CAB+RPV LA Q8W vs Q4W	Virologically suppressed adults	High satisfaction and strong preference for the Q8W schedule.
Kityo (2024) [22]	Phase 3, RCT (CARES)	CAB+RPV LA Q8W	Virologically suppressed adults in Africa	Non-inferior to oral ART in African populations.
Han (2025) [23]	Phase 1, RCT	Cabotegravir ULA	Healthy volunteers	PK profile supports dosing every ≥4 months.
ATTACHMENT INHIBITORS				
Lataillade (2020) [25]	Phase 3, follow-up (BRIGHTE)	Fostemsavir + OBR	HTE with MDR-HIV	Durable viral suppression (60%) at week 96.
Llibre (2024) [26]	Phase 3, follow-up (BRIGHTE)	Fostemsavir + OBR	HTE with MDR-HIV	Long-term safety and immune recovery up to week 240.
Anderson (2022) [27]	PRO analysis (BRIGHTE)	Fostemsavir + OBR	HTE with MDR-HIV	Significant improvements in quality of life.
Clark (2024) [28]	Biomarker analysis	Fostemsavir + OBR	HTE with MDR-HIV	Reduction in markers of inflammation and coagulopathy.
MATURATION INHIBITORS				
Spinner (2022) [30]	Phase 2a, proof-of-concept	Zabofiravir (monotherapy)	ART-naïve adults	Dose-dependent antiviral activity.
Pene Dumitrescu (2021) [31]	Phase 1, DDI study	Zabofiravir + Dolutegravir	Healthy volunteers	No clinically relevant pharmacokinetic interactions.
BROADLY NEUTRALIZING ANTIBODIES (bNAbs)				
Mendoza (2018) [33]	Proof-of-concept	Dual bNAbs (3BNC117+10-1074)	Suppressed adults (ATI)	Maintained viral suppression in the absence of ART.
Gaebler (2022) [34]	Clinical trial	Dual bNAbs (3BNC117+10-1074)	Suppressed adults (ATI)	Prolonged viral suppression (>20 weeks) in 76% of participants.
Sneller (2022) [35]	Clinical trial	Dual bNAbs (3BNC117+10-1074)	Suppressed adults (ATI)	Maintained suppression if baseline virus was bNAb-sensitive.

Julg (2024) [36]	Phase 1/2a, open-label	Triple bNAbs	Suppressed adults (ATI)	83% maintained suppression for at least 28 weeks.
Stephenson (2021) [37]	Phase 1, RCT	PGT121 (bNAb)	Viremic adults	Viral load reduction and long-term suppression in 2 cases.
Gunst (2023) [38]	Phase 2a, RCT (TITAN)	bNAbs +/- TLR9 agonist	Suppressed adults (ATI)	bNAbs delayed viral rebound; TLR9 agonist added no benefit.
Gruell (2022) [39]	Phase 2a, RCT (ROADMAP)	bNAb + Romidepsin (LRA)	Suppressed adults (ATI)	No clinically significant delay in viral rebound.
Corey (2021) [40]	Phase 2b, RCT (AMP)	VRC01 (bNAb) (PrEP)	At-risk adults	Highly effective (>75%) only against sensitive HIV strains.
Eron (2024) [41]	Phase 1b, RCT	Dual bNAbs + Lenacapavir	Suppressed adults (ATI)	Regimen was safe and maintained suppression for 26 weeks.
OTHER MECHANISMS & STRATEGIES				
Zang (2024) [42]	Phase 1, open-label	Islatravir	Healthy volunteers	Long intracellular half-life supports infrequent dosing.
Correll (2023) [43]	Post-hoc analysis	Islatravir + Doravirine	ART-naïve adults	Dose-dependent decrease in lymphocyte counts with ISL.
Molina (2024) [44]	Phase 3, RCT	Doravirine/Islatravir 0.75mg	Suppressed adults (switch)	Non-inferior, but CD4 decline halted development of this dose.
Mills (2024) [45]	Phase 3, RCT	Doravirine/Islatravir 0.75mg	Suppressed adults (switch)	Non-inferior, but CD4 decline halted development of this dose.
Landman (2022) [46]	Phase 3, RCT (QUATUOR)	ART 4 days on / 3 days off	Suppressed adults	Strategy was non-inferior to continuous daily therapy.
CURE STRATEGIES / PLATFORMS				
Young (2023) [47]	Preclinical study	Cabotegravir implants	Macaques	Protection against rectal SHIV infection.
Patel (2025) [48]	Preclinical study	Rilpivirine nanoparticles	Humanized mice	Drug concentration in myeloid cells.
SenGupta (2021) [49]	Clinical trial	Vesatolimod (TLR7 agonist)	Suppressed adults (ATI)	Modest delay in viral rebound.

Kessing (2017) [50]	Preclinical study	Didehydro-cortistatin A	Humanized mice	Suppression of viral rebound ("block-and-lock" strategy).
SYSTEMATIC REVIEWS / OVERVIEWS				
Flexner (2021) [1]	Narrative review	Long-acting drugs	General	Overview of LA drug development.
Łupina (2025) [2]	Systematic review	ART advances	General	Transition from oral ART to LA and immune therapies.
Noe (2025) [3]	Narrative review	Current and future LA options	General	Perspectives and unmet needs in LA therapies.
Thavarajah (2024) [32]	Systematic review	Use of bNAbs	General	Overview of the current state of bNAbs in treatment & prevention.
